# Tourism Environmental Carrying Capacity Review, Hotspot, Issue, and Prospect

**DOI:** 10.3390/ijerph192416663

**Published:** 2022-12-12

**Authors:** Cheng Long, Song Lu, Jie Chang, Jiaheng Zhu, Luqiao Chen

**Affiliations:** School of Environmental and Geographic Sciences, Shanghai Normal University, Shanghai 200234, China

**Keywords:** tourism environmental carrying capacity, sustainable development, Citespace, Vosviewer, Scopus

## Abstract

With the ongoing expansion of tourism, a conflict has arisen between economic growth in the tourism industry and environmental preservation, which has attracted the interest of government and academic groups. Because it enables the adaption of tourist activities and buildings in the tourism area in order to protect the natural resources of the scenic area while seeking economies of scale, the tourism environmental carrying capacity system is an essential tool for resolving this conundrum. It also enables tourist sites to grow sustainably while understanding their limitations and carrying capacity. This study uses Citespace 6.1.2 and VOSviewer 1.6.18 analysis software to conduct a bibliometric analysis and review of 297 articles on tourism environmental carrying capacity. This analysis includes early warning studies, assessment models and management tools, and analyses of keyword co-occurrence and emergent word co-occurrence. The article’s conclusion makes recommendations for further research, including the division of each interest group, improved dynamic forecast and early warning of tourism environmental carrying capacity, and the development of an objective, scientific model of tourism carrying capacity.

## 1. Introduction

The effects of tourism have been more and more prominent in conversations and studies about development during the last few decades. The tourism industry has a huge potential to spur economic development in destinations. However, its expanding effects have resulted in a number of current and potential issues, as well as environmental, social, cultural, economic, and political problems in destinations and systems that call for alternative, more environmentally and destination country-friendly approaches to development, planning, and policy. In the early stages of tourism growth, there is a far more supply of newly created destination regions’ resources, facilities, etc., compared to the demand from travelers, who can make use of the destination’s many tourism resources and surroundings. While the growth of tourist destinations, commercial advertising, word-of-mouth marketing, and other methods to increase the popularity of the destination, its development into an upward stage, followed by a significant influx of tourists, although during this period, the destination will also strengthen the corresponding supporting facilities and improve the upgrade, etc., the supply measurement end of the increase may be significantly less than the demand side. Additionally, some tourists’ moral character may lead them to litter, paint on the walls, destroy display cabinets and signage, and other actions that are not suited to the development of tourism sustainably, such as the depletion of resources, pollution of the environment, damage to facilities, and customer dissatisfaction. The measurement of tourism environmental carrying capacity (TECC) is a potent tool to achieve this goal and will be crucial in scenic areas and tourist destinations. Thus, it is vital to increase the control and dynamic monitoring of the destination.

The TECC, initially known as tourism volume, refers to the threshold of the intensity of tourism activities that the natural, economic, and social systems of a tourism destination can withstand, and it is essentially a comprehensive reflection of the structural characteristics of the tourism environment system. In 1964, American scholar J. Alan Wagar published his academic monograph “Carrying Capacity of Wildlands for Recreation” [1]. According to Wagar, recreation capacity refers to the amount of recreation used in a recreation area that can maintain tourism quality in the long term. Since Wagar, TECC research results have been emerging. The World Tourism Organization first used the phrase “tourism capacity” in its report work from 1978 to 1979, which officially introduced the concept to the world of international research [2]; then, scholars and various stakeholders reached a consensus on its important role in the conservation of natural systems which plays an important role in sustainable tourism. In February 1987, at the 8th World Commission on Environment and Development held in Tokyo, Japan, *Our Common Future* was adopted and subsequently published, pointing out the importance of sustainable development for the common destiny and common future of humankind and the concept of sustainable development was then introduced into tourism research and policy [3], and sustainable tourism and TECC have attracted considerable interest from tourism researchers, including the establishment of a thematic journal, *Journal of Sustainable Tourism*, which now has an impact factor of 9.37.

At the UN Sustainable Development Summit held in New York on 25 September 2015, the 193 member states of the United Nations formally adopted 17 Sustainable Development Goals (SDGs), abbreviated as SDGs [4]. The SDGs aim to shift to a sustainable development path by thoroughly addressing the three dimensions of development—social, economic, and environmental—in an integrated manner from 2015 to 2030. Among them, the 14th and 15th are protecting and sustainably using oceans and marine resources for sustainable development; protecting, restoring, and promoting sustainable use of terrestrial ecosystems; sustainable forest management; combating desertification, halting and reversing land degradation, and curbing biodiversity loss, respectively. Currently, human activities such as pollution, fishery depletion, and habitat loss are thought to have “severely damaged” up to 40% of the world’s oceans. In addition to providing food security and protection, forests—which cover one-third of the earth’s surface—are important for halting climate change, preserving biodiversity, and housing indigenous peoples. 13 million hectares of forest are lost each year, and 3.6 million hectares of land get desertified as a result of ongoing dryland degradation. The livelihoods and attempts of millions of people to escape poverty are impacted by deforestation and desertification brought on by human activity and climate change, which represent serious obstacles to sustainable development. Both the sea and the land are important hosts for tourism activities. In terms of the marine aspect, there is the coastal tourism of the Gold Coast, the sunny beaches of Catalonia, the Great Barrier Reef of Australia, the sea surfing of California, the Scandinavian fjord scenery, the sunny coast of Providenciales in the Turks and Caicos Islands of the Caribbean, etc. The terrestrial aspect includes Yellowstone National Park, the Koktokay Global Geopark, Sipsongpanna Rainforest, Kenya Wildlife Reserve, Masai Mara Savannah, etc. The majority of these well-known tourist destinations are mature tourist destinations, and all of them are struggling with overloading. As a result, their corresponding marine or terrestrial environments will suffer accordingly, necessitating a stronger study of the carrying capacity of the environment that is not just focused on the aforementioned tourist destinations.

The TECC has been the subject of numerous theoretical and empirical studies (Table 1), but the research in this area is still in need of systematic trawling. Additionally, almost all of the studies use the CNKI and Web of Science databases as their primary literature sources rather than the more comprehensive Scopus database. The purpose of this paper is to analyze the research progress, theoretical concepts, assessment models, management tools and early warning systems of TECC, especially how to recognize and set the environmental carrying capacity of a tourism destination, and to propose a clear structure for subsequent research through the review. Although the TECC of land-based and sea-based tourism destinations must be very different, this paper uses conceptual research to explore the improvement direction and coupling points for subsequent research on the topic of TECC. The subsequent structure of this paper is as follows. Section 2 describes the analytical methods used in the study and the sources of research data; Section 3 presents the analysis results, including bibliometric analysis, keyword co-occurrence and emergent word co-occurrence analysis, and also reviews and summarizes the related conceptual studies, assessment model studies, applied studies and management tools and early warning studies, and makes a research review; the Section 4 final discussion and Section 5 conclusion section summarizes the findings of the article and presents the limitations of the article and future research prospects.

## 2. Methodology

In this study, the Mapping Knowledge Domain (MKD) was used [20] to analyze the scientific research results of the TECC from 1982 to 2022. In terms of the research idea, it follows the strategy from macro to micro, from whole to local, and from intuitive simplicity to in-depth complexity [21].

### 2.1. Mapping the Knowledge Domain

As a cutting-edge method in the field of scientometric analysis technology, knowledge domain mapping combines the theories and methods of applied mathematics, information science, computer science and graphics with the methods of co-citation analysis and co-occurrence analysis in bibliometrics, using knowledge mapping to demonstrate the core structure, development history, frontier areas and knowledge framework of the discipline. It solves the problems of traditional literature research methods, such as difficult data screening and heavy workload and has the advantages of being scientific, comprehensive, standardized, accurate and simple [22]. In this study, CiteSpace 6.1.2 and VOSviewer 1.6.18 software were used to map the knowledge domain of TECC.

### 2.2. Collection of Literature Data

Scopus is a multidisciplinary abstract-indexed database launched by Elsevier [23] in 2005 (http://www.scopus.com, accessed on 10 September 2022), which now uniquely combines a comprehensive, expertly curated abstract and citation database with enriched data and linked scholarly literature across a wide variety of disciplines. In Scopus, scholars can quickly find relevant and authoritative research, identifies experts, and get access to reliable data, metrics, and analytical tools. Boasting the largest pool of author profiles available (17 million and counting), Scopus easily outmatches the competition [24]. It is one of the standard citation, bibliometric and abstract databases in the field of scientometrics and bibliometrics. Scopus has significant advantages over the Web of Science database in terms of reference completeness, indexing, and researcher relations [25].

First, in order to obtain as much relevant literature as possible, “Tourism Carrying Capacity”, “TECC”, and “TCC” were used as keywords in the “Topic” of the literature search, including title, abstract, and keywords; second, the time span was set to “1982–2022”, and then the search code was set to (TS = Tourism Carrying Capacity, TS = TCC or TS = TECC) and Language: (English) and Time range: (1982–2022). The search was conducted on 25 July 2022, and the database was last updated on 5 August 2022. A total of 862 results were collected and carefully checked (Since there are other research themes also abbreviated as TECC or TCC, e.g., Tactical Emergency Casualty Care, Technology Commercialization Centers, and Transnational Capitalist Class. We have then undergone a two-round screening). In the first round of screening, monographs, conference proceedings, and book reprints were excluded, and only journal articles and reviews in English-language were collected, as these are generally considered more influential and reputable than the formers. In the second screening round, results that did not involve tourism, travel, or leisure studies and were not related to environmental carrying capacity were excluded from this study. Finally, 297 valid re-study results were retained as the literature sample.

### 2.3. Analysis Methods

To address the objectives of this study, the article uses analysis methods such as keyword co-occurrence and bursty analysis and analyzes the volume of publications and journal trends.

Co-word analysis uses the co-occurrence of word pairs or noun phrases in a collection of literature to determine the relationship between topics in the discipline represented by that collection. Keywords are the core summary of a paper, and analysis of keywords in a paper can provide a glimpse into the topic of the paper [26]. Several keywords given in a paper must be related in some way, and this association can be expressed in terms of the frequency of co-occurrence. It is generally believed that the more frequently the word appears in the same paper, the closer the relationship between these topics.

Bursty words are keywords with low frequency but increasing growth momentum [27]. This indicates that the keyword is receiving more and more attention from scholars in the subject area and has a higher probability of developing into a research hotspot in the future [28]. The development of things follows the basic life cycle theory, and keywords are no exception. Generally speaking, there are four stages of keyword development in the process of scientific communication: emerging, developing, maturing, and diminishing. Burst topic detection works especially well in online social media and burst word detection is a significant issue in the field of information metrics study internationally.

CiteSpace and VOSviewer software can be used to analyze a large number of keywords for co-occurrence and burst detection and to identify hot spots and trends in a scientific field more objectively and effectively.

The network construction in CiteSpace is based on time slice. In this study, the unit of the time slice is set to 5 years (i.e., a total of 8-time slices). Note types are selected as Author, Institution, Keyword and Reference for analysis. The network construction in VOSviewer is based on the whole, and in this study, the Type of analysis selects Co-occurrence and Bibliographic coupling, respectively, and the Unit of analysis selects All Keywords and Sources, respectively.

## 3. Results

### 3.1. Overview

**Figure 1:**
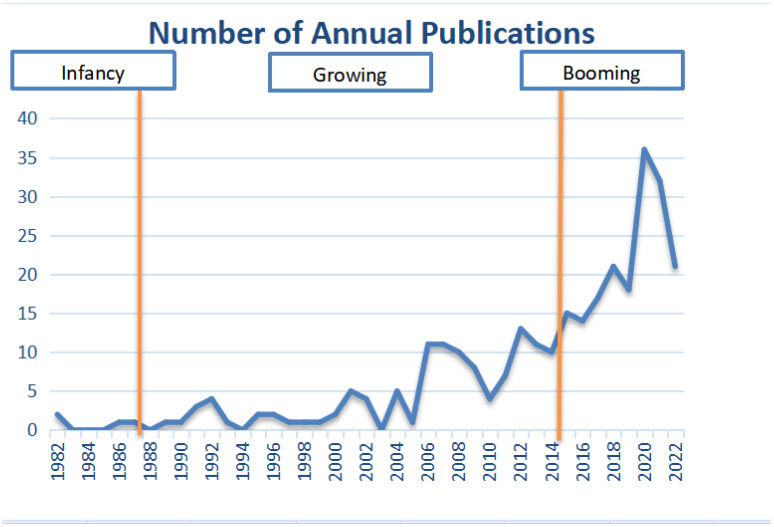
Annual Publication statistics of TECC during 1982–2022.

The annual distribution of literature is a mapping of the quantity of literature in the time dimension. It is a quantitative basis for understanding the research progress and classifying the research stages and can reflect the development level of TECC research to a certain extent. Figure 1 shows the number of works of literature in the research sample, with an overall increasing trend, indicating that scholars pay more and more attention to the research on TECC. In general, scholars’ attention to TECC can be divided into 3 stages. The first stage was before 1987, TECC was in its infancy, and very few keen environmental scholars paid attention to the environmental carrying capacity problem under the theme of tourism. The number of articles issued in this stage is only sporadically distributed; the second stage is the middle growth stage from 1987 to 2015 since the adoption and publication of *Our Common Future* at the 8th World Commission on Environment and Development in Tokyo, Japan, the concept of sustainable development was introduced into tourism research and policy at this point, and the theme of TECC received significant attention. Along with environmental issues or overload issues in some tourist destinations, etc., many empirical studies of TECC were conducted at this time. The third stage is the booming stage from 2015 to the present, with the official adoption of 17 Sustainable Development Goals (SDGs) at the United Nations Sustainable Development Summit held in New York in 2015; as all sectors pursue SDGs, researchers also work toward sustainable development, and since the travel and the tourist industry is a key component, sustainable tourism development has become a hot topic. They tend to focus on projects that are relevant to the TECC. The number of articles published has reached a peak in the last two years. Conceptual studies, assessment model studies, management tools, and early warning studies, as well as empirical studies on the TECC of various types of destinations, have been flourishing and appearing in various journals.

**Figure 2:**
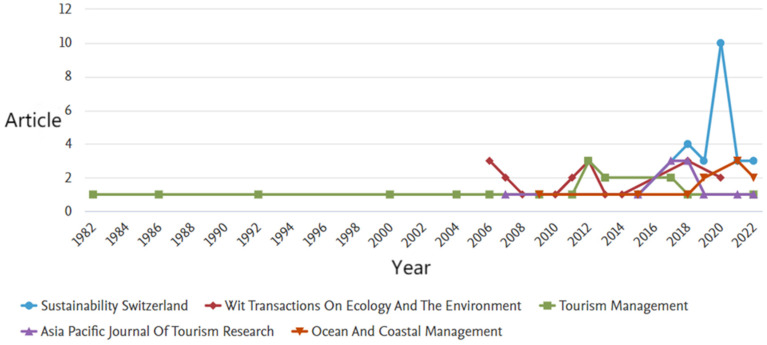
Top 5 journals in terms of articles on the TECC.

**Figure 3:**
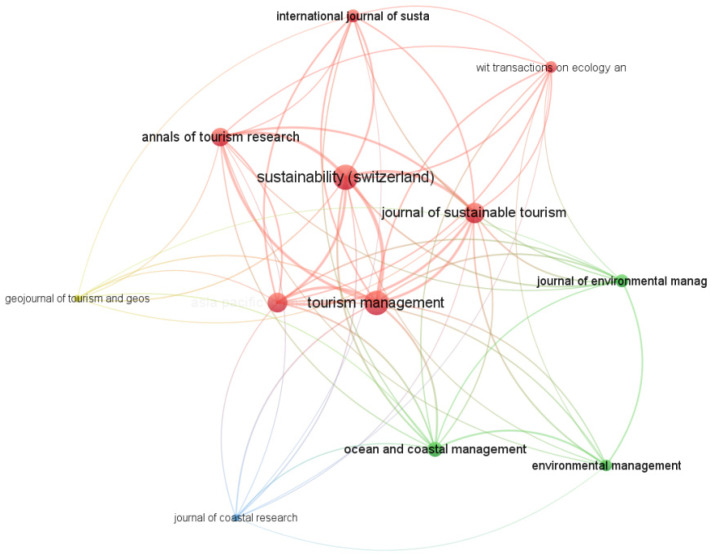
The coupling relationship between journals on the theme of TECC.

As is shown in Figure 2, the top 5 journals in terms of the number of articles published are Sustainability, Wit Transactions on Ecology and The Environment, Tourism Management, Asia Pacific Journal of Tourism Research, and Ocean And Coastal Management, among which, Sustainability has 24 articles. In contrast, Tourism Management was early to focus on the topic of TECC, maintaining a small number of articles in the 1980s, while the other journals lacked this research tradition. Almost all of them only started to get involved in this topic after 2005, and even though the number of articles is relatively high, the theoretical foundation seems to be less solid than that of Tourism Management, which, of course, must be admittedly related to the time of the creation of each journal. It is worth noting that, as one of the SDGs’ sustainable development goals, i.e., the conservation and sustainable use of oceans and marine resources for sustainable development, the TECC in the ocean has received a great deal of attention from scholars, and Ocean and Coastal Management is a journal that covers most of the authoritative articles on the TECC in marine areas. In addition, the International Journal of Sustainable Development and World Ecology, the Journal of Sustainable Tourism, and Environmental Management are also important journals for TECC research (Figure 3.).

### 3.2. Keyword Co-Occurrence Analysis

**Figure 4:**
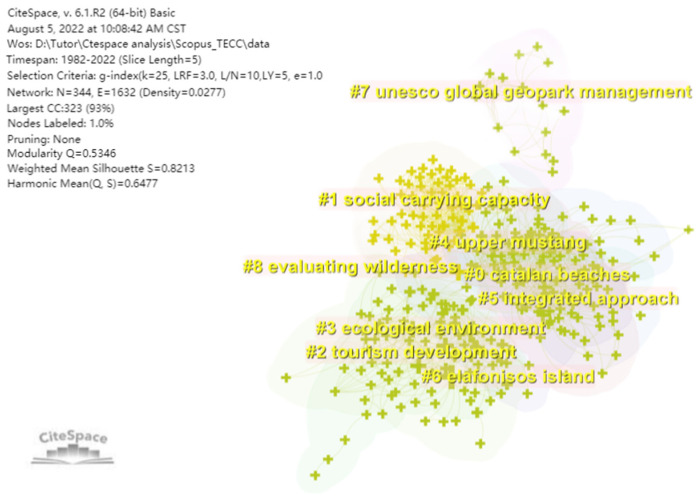
Keyword co-occurrence analysis with 5 years as a time slice.

**Figure 5:**
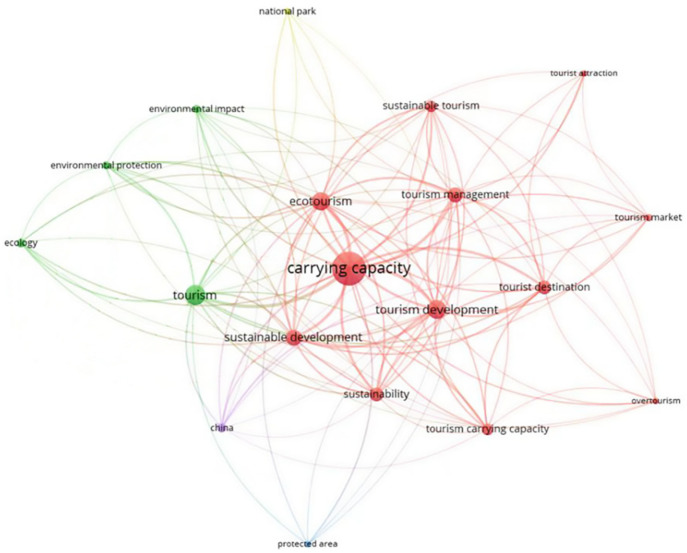
Overall keyword co-occurrence analysis.

From the results of the keyword co-occurrence analysis presented in Figure 4, the earlier studies (#7 #8) focused on the TECC measurement of natural tourism destinations, including global geoparks [29], national parks [30,31], recreational wetlands [32] and independent islands [33] (#6), while subsequent studies (#5) have sought to change the traditional way of measuring the TECC [34] and exploring integrated measurement methods. In the 21st century, research on TECC is no longer limited to the measurement of physical carrying capacity but also focuses on the impact of variables such as destination perception, tourist satisfaction and resident perception on tourism carrying capacity and introduces social carrying capacity [35,36] (#1). In 2015, with the United Nations Sustainable Development Summit held in New York, scholars increased their attention to the sustainable development of the ocean, and in terms of marine tourism, Catalonia, Spain, has the most abundant tourism resources and is the most popular marine tourism destination, with a large number of tourists from the United Kingdom, Northern Europe and North America throughout the year all seasons, and the study of the environmental carrying capacity of this region has become a recent hot topic [37,38] (#0). As a whole, sustainable development, destination management and ecotourism are the main classical topics in TECC research (Carrying Capacity and research topics are similar and not distinguishable, so they are excluded). It is noteworthy that the keyword China, which is the only geographical category keyword to appear in both two keyword co-occurrence analyses (Figure 5.). Despite the fact that China’s tourism research started late, it has already resulted in a significant number of articles on the TECC due to the country’s abundant tourism resources and a sufficient number of visitors, as well as the contradictory issues between visitors, tourism resources, and residents, which have drawn attention from many scholars.

### 3.3. Co-Occurrence Analysis of Bursty Words

**Figure 6:**
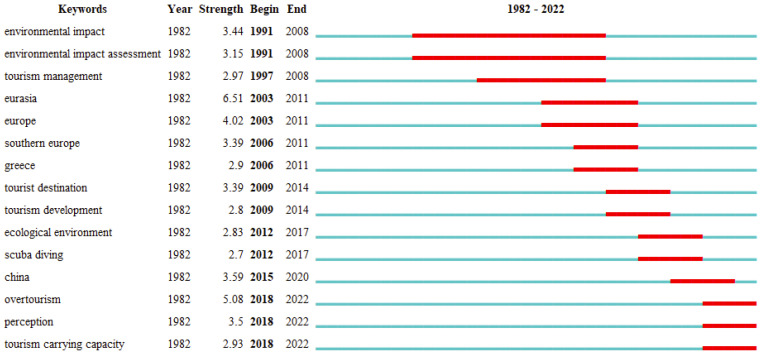
Co-occurrence analysis of emergent words (with a 5-year time slice).

As a complement to the keyword co-occurrence analysis, the bursty word analysis demonstrates how the study hotspots for TECC have changed over time (Figure 6.). Since the 1990s, overtourism has had a negative impact on the growth of some tourist sites, especially in terms of irreparable environmental damage. From 1991 to 2008, environmental impact assessment has become a popular topic. When it comes to regional differences, the European region is well-known as a travel destination because of its abundant tourism resources, earlier tourism development, and more developed destination system, while the Asian region is well-known as a travel destination because of its proximity to the European market and distinctive tourism resources, which draw travelers from all over the world [39]. Therefore, in the period 2003–2011, Europe, Eurasia, Athens and Southern Europe were the focus of research on the TECC in the period 2006–2011 [40]. The rise of scuba diving has been more than a decade, and as a highly participatory tourism experience, it has had a great impact on the marine environment, marine biodiversity (fish habitats, coral reefs), etc. The study of the location and reasonable capacity of reasonable areas for scuba diving is a popular theme for 2012–2017 [41,42]. In recent years, with the new crown epidemic for social distance, many tourist destinations are in the stage of overtourism, and while re-measuring the environmental carrying capacity, scholars have also noted the changes in the perception of tourists and residents, introducing the social carrying capacity [36]. Thus, perception and overtourism are the hot spots of recent studies. Notably, TECC research in China was also a major hotspot during the period 2015–2020 [43,44]. However, as a result of COVID-19 in 2020 and the exponential drop in foreign travelers, study on this topic has slowed down.

### 3.4. Literature Review of Conceptual Studies, Assessment Model Studies, Applied Studies, Management Tools, and Early Warning Studies

Neo-Malthusianism gave rise to the idea of TECC, which later developed into a comprehensive system that includes physical capacity, tourist thresholds, growth management estimates, etc. Numerous research on the environmental impact of tourism has been conducted, but their development has not been systematically sorted out. Various assessment models have been developed from the first descriptive and straightforward data used in these studies. However, it is debatable if various models can adapt to various settings and circumstances. Additionally, there are multiple kinds of tourist destinations, and these destinations exhibit different characteristics and experience varying degrees of overload issues. In order to address these issues and support the tourism destinations’ sustainable development, management tools and early warning systems must be used. As a result, the conceptual analysis of the TECC, the study of assessment models, the empirical analysis of categorized destinations, and the analysis of management tools and early warning systems constitute the four main components of this chapter’s literature review. According to these four results, we further conclude 4 characteristics of current research and put forward 4 prospects regarding the future research agenda.

#### 3.4.1. Conceptual Research

The concept of recreational carrying capacity evolved from a neo-Malthusian perspective of resource limitation. The concept of carrying capacity was originally developed in the field of range and wildlife management, based on the notion that organisms can only survive within a limited range of physical conditions, i.e., “the availability of suitable living conditions determines the number of organisms that can exist in the environment” [45], the problem faced by these fields relates to the physical capacity of a given area of pasture, hay-field or heathland to maintain the quantity and quality of forage over time to sustain a given number of domestic or wild livestock. The TECC evolved from the environmental capacity; in 1963, W. Lapage [46] first proposed the concept of TECC based on the study of the maximum capacity of the tourism environment. In 1964, American scholar J. Alan Wagar [1] published his academic monograph *Carrying Capacity of Wildlands for Recreation*. He argues that recreation capacity is the amount of recreation used in a recreation area that can sustain tourism quality over time. Research on tourist capacity has developed since Wagar. The term tourism capacity was formally introduced by the World Tourism Organization in its work report in 1978–1979, marking the beginning of tourism capacity into the scope of international research. Hovinen (1982) [47] defined carrying capacity as the maximum number of tourists that can be accommodated without causing excessive environmental degradation and without leading to a decrease in tourist satisfaction. Mathieson and Wall (1982) [48] defined carrying capacity by considering the physical impact of tourism on a destination in terms of environmental and experiential aspects, such as the maximum number of people who can use the recreational environment without an unacceptable decline in the quality of the recreational experience. On the other hand, O’Reilly (1986) [49] described two schools of thought on carrying capacity. One, carrying capacity is considered to be the ability of a destination area to absorb tourism before the negative effects are felt by the host and resident. Carrying capacity is determined by how many tourists are wanted, not by how many tourists can be attracted. The second view is that TECC is the level of tourist flow that is exceeded because some of the tourists’ own perceived capacity has been exceeded, and therefore the destination area no longer satisfies and attracts them. O’Reilly (1986) [49] also points out that Mathieson’s definition only considers the physical impact of tourism on the destination from an environmental and experiential perspective. He claims that carrying capacity can be established not only from a physical perspective but also for the social, cultural and economic subsystems of the destination. As described by Mathieson, economic carrying capacity is the ability to absorb tourist functions without crowding out desirable local activities. They define social carrying capacity as the degree to which the host and resident of an area become intolerant of the presence of tourists. Lindsay (1986) [50], in discussing the TECC of national parks, defined it as the physical, biological, social, and psychological capacity of the park environment to support tourism activities without degrading environmental quality or visitor satisfaction. Reilly [49], on the other hand, argued that the perceived condition of residents in tourist destinations is an important factor affecting the carrying capacity of the environment and that the psychological perception of residents directly affects the carrying capacity. In 1995, Cui [51] proposed to use the TECC instead of the tourism environmental volume and defined it as “the number of tourists that a destination can bear in a certain period of time under the premise that the current situation and structural combination of a tourist environment (i.e., tourism environmental system) do not change in a harmful way to the present and future people”. The study of TECC is conducive to promoting the implementation of environmental protection policies and promoting rapid regional economic growth. Jovic (2009) [52] argued that TECC is the maximum number of tourists that can stay in a given area without causing unacceptable and irreversible changes in the environmental, social, cultural and economic structure of the destination and without reducing the quality of the tourism experience. Zelenka and Kacetl (2014) [53] pointed out that the TECC is not only a matter of the number of visitors to a destination. Rather, it is also related to a range of other factors, including infrastructure, tourism distribution, and visitor behavior patterns, and may focus on specific dimensions in each particular geographic context. Therefore, the assessment of the TECC can also be done in many different ways and reflected in different dimensions such as physical, sociocultural, and economic development dimensions. Kisiel et al. [54] conducted an in-depth theoretical discussion and proposed that TECC should include two aspects: first, the natural environmental capacity, and second, the perceived environmental capacity, which is the ability to accommodate tourists on the basis of ensuring a good tourism experience. Milla et al. [55] determined the connotation of TECC and established the definition of TECC. It is believed that TECC should include indicators of four aspects: natural environmental carrying capacity, the spatial carrying capacity of resources, economic carrying capacity, and psychological carrying capacity. These tourism environmental carrying capacities, measured based on different dimensions, show that TECC is a complex system. The related research has shifted from discussing reasonable quantity to growth management and optimal decision-making objectives.

#### 3.4.2. Evaluation Model Research

In the early studies of TECC, scholars mostly used descriptive and simple statistical methods. However, with the development of tourism, science and technology, these methods have become outdated. Currently, there are two types of TECC prediction: quantitative studies and qualitative studies, of which the former is the most commonly used research method. Han [56] established a linear planning model of low carbon TECC through a fuzzy linear function with “tourism scale economy” as the objective function and the constraints of resources and ecological environment factors as the constraints, studied the TECC in Shandong Peninsula and Sanya City, China. Ye et al. [57] used a state-space model to construct a TECC early warning index system from natural, economic and social aspects, explored the current situation and spatial and temporal differences of TECC early warning in 10 island cities in eastern China, and used BP (Back-Propagation), neural network model, to predict the development trend of early warning. Wang et al. [58] constructed a utility theory framework of TECC based on consumer utility theory and calculated the TECC thresholds under different environmental conditions in the park using the conditional logit model. Tokarchuka [35] used subjective well-being theory to analyze the social carrying capacity of tourism in Berlin 12 by the regression model. Wang [59] analyzed the TECC of Emei Mountain with empirical modal decomposition and BP neural network as a new method of predicting TECC by government staff and scenic area managers. Yan [60] studied the TECC in East Lake scenic area by fuzzy hierarchical analysis and set-pair analysis. Chen [44] measured the TECC at the county level in Zhoushan Islands by ecological footprint quantification. Alvara [38] studied the TECC of the Catalan coast using input-output analysis, which allows to distinguish economic flows within different spatial units, including direct, indirect and induced effects, and to quantify spillover effects, which are usually significant in the tourism industry. Gonzalez and David [36,61], using ANOVA, studied the TECC in the small town of Besalou, Spain, and the coral reef area of Etla, northern Red Sea. Mark T [62] compared the TECC of two resorts in Papua New Guinea and Mexico with energy analysis. Jurado E [63] created two synthetic indices (weak and strong) using the DPSIR model and the GIS-MCDA method to analyze the carrying capacity of the eastern Costa del Sol in Spain. Adamchuk [64] developed a quantitative evaluation model of the integrated carrying capacity of scenic areas based on the product matrix vector length method and obtained a favorable measure of the integrated carrying capacity of scenic areas. Cvijanovi’c et al. [65] used the theoretical speculation method and empirical measurement method to construct the measurement formulae of ecotourism environmental capacity, natural resources environmental capacity, tourism space environmental capacity, social ecotourism environmental capacity and tourists ecotourism environmental capacity. Mohanty et al. [66] analyzed the cumulative effect of tourism activities on environmental capacity and established a formula for calculating TECC using quantitative relationships of environmental factors and Pareto optimality. Kalchenko et al. [67] proposed a model for measuring the LECC with length, area and recreational facilities as limiting factors and measured the TECC through the design of the model. Meanwhile, Shia [68] also calculated the TECC of Shangri-La county in China by area method. In the process of quantification, the TECC index system is influenced by value judgments, and in the absence of specific criteria of “management objectives” or “ideal conditions”, although the study of TECC emphasizes scientificity, objectivity and accuracy, its measurement model is full of problems, the measurement model is full of subjective factors.

#### 3.4.3. Application Research

In the empirical study of TECC-categorized destinations, it presents a shift from initially fragmented point-like to tourist routes and county-based tourist destinations, etc., from point to line and surface, and the research scope coexists with micro-scale, mesoscale and macro-scale. Scholars have explored TECC based on different theoretical perspectives, including DEPSIR [69], PSR [70] and EES [71] models. The local TECC studies include national parks [58], tourist resorts [62], lakes [60], islands [72], forests [73] and coast regions [63], etc. Scholars analyzed the TECC based on first-hand surveys and measurements and combined it with secondary data; then extended to archipelago [44], counties [68,74], cities [35,75] and specific regions [56,76]. The TECC measurements are mostly modeled and quantitatively analyzed using national or local statistical yearbook data and panel data. Despite the fact that there are many studies on the various types of destinations for TECC, almost all of them are self-adaptive studies based on the respective destinations, and the corresponding comparative studies are scarce. Plus, these studies are weak in extension and expansion regarding co-research, which undermines the systemic nature of TECC theme studies.

#### 3.4.4. Management Tools and Early Warning Research

While foreign scholars call for national laws to guarantee the role of management tools, domestic scholars place more emphasis on improvement-oriented management initiatives in tourism destinations. Common management tools include the limits of acceptable change (LAC) [77], visitor experience and resource protection (VERP) [30], visitor activities management process (VAMP) [78], visitor impact management (VIM) [79], recreation opportunity spectrum (ROS) [80], and tourism optimization management model (TOMM) [81], etc.

The research on TECC early warning system is in the preliminary exploration stage, and current research mostly focuses on wetland parks [57], marine parks [82], and the carbon cycle [83], which are in a broad sense of natural resources [84]. In terms of early warning models and research methods, they basically adopt methods consistent with the carrying capacity. Some scholars have done further research by improving the existing assessment methods, but most of them stay in the early warning situation of a single method without a breakthrough. Its theoretical and methodological system is still immature. Current research is basically based on relevant statistical and econometric methods, and scholars mostly subdivide the TECC into multiple subsystems as the analysis framework, such as Huo [85] based on the large system theory divides the tourism early warning system into subsystems such as tourism alarm dynamic monitoring, tourism alarm source analysis, tourism alarm sign identification, tourism alarm degree forecast and geographic information technology assistance. Zhao [86] established a regional tourism ecological security composite early warning system consisting of a regional tourism ecological environment pressure warning subsystem, a regional tourism ecological environment quality early warning subsystem, and regional tourism ecological protection and remediation capacity early warning subsystem. The current research tries to construct some early warning index systems, but mostly from qualitative research and time interface analysis based on empirical and historical data, lacking in-depth research on the regional differences and temporal changes of TECC, which substantially reduces its early warning effectiveness and significance.

### 3.5. Research Review

From the current research results, the study of TECC generally presents the following characteristics: (1) The differentiation of conceptual research. The scholars have different interpretations regarding the concept of TECC and make its research content very different, including but not limited to the measurement of the natural environment, resource space, social-economic, cultural and psychological aspects, but reach a consensus on its purpose, including promoting the sustainable development of the destination and enhancing the satisfaction of tourists and residents. (2) Continuous enrichment of assessment models and deepening of research methods. Assessment models have shifted from qualitative descriptions and simple statistical methods to modeling analysis and related methods, including non-causal time series models, causal relationship models, artificial intelligence models and combination models, and quantitative measurements with BP neural networks, fuzzy hierarchical analysis, set-pair analysis, input-output analysis and area method, etc., and research results have become more objective and reasonable. (3) Gradual expansion of research scale. The initial empirical research of TECC focused on some micro-regions such as Colorado Grand Canyon National Park [30], Red Sea Coast [61], and the Alcatraz Islands [33], etc., and then expanded to larger-scale studies, including Shangri-La County [68], Shandong Peninsula City Cluster [56], the city of Berlin [35], Japan [87], and the Maldives [88]. The study of TECC shows a trend of turning point-line-surface. (4) The management tool system has been improved. Although the research on management tools and the early warning system of TECC is still in the initial stage, scholars have combined with the theory of tourism early warning system and put forward a series of management tools, including LAC, VERP, VAMP, VIM, ROS and TOMM, etc. Based on natural resources, tourists’ and residents’ satisfaction perspectives, they have established a system of “indicators” reflecting the quality of tourism experiences and resource conditions, established “standards” for minimum acceptable conditions, proposed “monitoring techniques” for timely and appropriate management tools to ensure that the state of the corresponding areas meets these standards, and developed “monitoring techniques” to ensure that the conditions of the various areas meet these standards. The management measures” to ensure that the various indicators are maintained within the specified standards have been developed.

Based on the characteristics summarized in the above discussion, this paper puts forward the following outlook for the subsequent research on TECC: (1) strengthen the growth management and optimal decision target research of TECC. TECC is a complex system, and it being measured based on different dimensions shows that natural resources, economic and social, cultural and psychological subsystems are all factors involved in TECC, and future research should focus on the dynamic evolution of TECC and can simulate and predict TECC through the neural network, machine learning and other methods to reduce the measurement model in subjective factors to achieve the goal of optimal dynamic decision-making. In addition, it is necessary to adjust according to the empirical object and carrying capacity management objectives and combine the relevant theories of sociology, psychology and economics to build a scientific and objective TECC model. Secondly, subdividing each stakeholder and conducting comparative research according to the needs of each stakeholder can be done with the help of coupling theory in order to achieve balance or maximize the comprehensive benefits of each interest subject. (2) Combined with the software of related disciplines for comprehensive analysis. TECC involves ecology, environmental science, geography and sociology and other disciplines, and the existing research only focuses on the use of a discipline of software for a single aspect of the measurement, which weakens the overall systemic TECC. Subsequent research can focus on integrated software such as Ansys Fluent, ENVI and ArcGIS for simulation prediction, forward inversion and spatial analysis to scientifically study the overall system of TECC. (3) Focus on the expansion of TECC research. Although the research on the TECC of various types of destinations is rich, the articles are almost all based on the characteristics of the destination for adaptive quantitative research. The corresponding comparative research is relatively small, and its extension and expansion are weak, weakening the systemic nature of TECC theme research. Future research should take into account the extended adaptive range of TECC while focusing on the ontological self-adaptation of case sites, which will make the research more theoretical and practically meaningful. (4) Research on succession management tools and early warning systems. In terms of time series, the existing research is increasingly inclined to find some reasonable and effective capacity management tools on the basis of a specific understanding of environmental capacity conditions so as to achieve the goal of capacity control and the development of capacity management tools has become a new hot spot for research. At the same time, on the basis of time interface analysis based on experience and historical data, deepen the research on geographical differences and time series changes of environmental carrying capacity to better realize the role and significance of management tools and early warning systems, future research can introduce neuro-tourism simulation experiments, scenic spot management simulation and tourism safety simulation experiments, etc., to strengthen the simulation for tourism places and TECC in order to realize dynamic control.

## 4. Discussion

### 4.1. Overview

In this paper, a bibliometric analysis of 297 articles retrieved from the Scopus data platform, including volume data, journal distribution, keyword co-occurrence and bursty word co-occurrence analysis, was conducted in Citespace and VOSviewer analysis tools, and the study found that.

1. Before the 21st century, the topic of TECC received less attention because most tourist places were in the early initial development stage. Since entering the 21st century, with the booming tourism industry and the emergence of some negative impacts in some tourist places, the research on TECC has increased greatly, especially in the last 5 years.
2. Sustainability, Wit Transactions on Ecology and The Environment, Tourism Management, Asia Pacific Journal of Tourism Research, Wit Transactions On Ecology And The Environment, Tourism Management, Asia Pacific Journal of Tourism Research and Ocean and Coastal Management are the international journals with the most articles on the topic of TECC, with 24 articles on Sustainability. In contrast, the Tourism Management journal was early to focus on the topic of TECC and has been published annually since the 1980s, although only about 1 article per year in the early years.
3. Early studies on TECC mostly focused on measuring TECC in natural tourism destinations, including areas such as global geoparks, national parks, recreational wetlands and independent islands, while subsequent studies focused on elements of tourist satisfaction and residents’ perceptions, with an eye on social carrying capacity and economic carrying capacity. In 2015, with the UN Sustainable Development Summit held in New York, marine sustainability became an important topic, especially in Catalonia, Spain, where the study of environmental carrying capacity has become a recent hot topic. In addition, the European region (especially Athens) and the Asian region have been the main regions of TECC studies in the last 20 years.
4. The concept of TECC originates from environmental carrying capacity, but in comparison, TECC contains various socio-economic and psychological factors, which is more complex, and a unified definition of this content has not yet been formed; the measurement methods of TECC have shifted from the initial descriptive and simple statistical methods to computers (such as BP neural networks), GIS and integrated models. However, the research on early warning and management tools of tourism carrying capacity is relatively less.
5. The study of TECC generally presents four characteristics, i.e., The differentiation of conceptual research, continuous enrichment of assessment models and deepening of research methods, gradual expansion of research scale and the improvement of the management tool system.

### 4.2. Shortcomings of the Article

Although the study reviewed articles on TECC and used literature data from the Scopus database for the bibliometric analysis to remedy some of the shortcomings of previous studies, there are some limitations in the article, such as the study only selected journal articles and review articles for the analysis, which may ignore some important and relevant literature. In addition, in the bibliometric analysis, only English-language literature was selected for the analysis, while regions such as Provence, Brazil, and Japan, which are some recent tourism hotspots, are non-English speaking areas, which can make the analysis results somewhat limited. Plus, TECC research in Western and Central European countries, especially in France, Germany, Italy, and Poland, started earlier than in North America, but those findings were most published in their national languages and are usually ignored. Nevertheless, this study provides a systematic review of TECC studies in the Scopus database for the period 1982–2022 to provide references for subsequent empirical studies and tourism destination management practices. Future studies could include conference articles and other language sources for analysis (e.g., Spanish and French) to compensate for the limitations of the current study and pay more attention to the early studies in Western and Central European countries to better close the knowledge gap.

## 5. Conclusions

Based on Citespace and VOSviewer analysis software, this paper conducts a bibliometric analysis and literature review on 297 articles screened from the Scopus database on TECC, including keyword co-occurrence and bursty word co-occurrence analysis and research on concepts, applications, assessment models and management tools and early warning, followed by a review of existing research, including the divergence for conceptual research, the continuous enrichment of assessment models and the deepening of research methods, the gradual expansion of research scales and the continuous improvement of management tools, and proposes four future research directions, namely, strengthening the growth management of TECC and optimal decision-making objectives, combining software from related disciplines for comprehensive analysis, focusing on the expansion of TECC research, and continuing the research of management tools and early warning systems. Finally, the findings of the bibliometric analysis and literature review are discussed, and the limitations of the paper are pointed out as well as the directions for remediation. Notably, early TECC studies were primarily published in non-English languages in Western and Central European nations, particularly in France, Germany, Italy, and Poland, and this may have led to a knowledge gap to some extent. Future studies should find an approximate solution to this challenge, either by making comparisons or by doing research on these corresponding national languages.

## Figures and Tables

**Table 1:** Summary of 11 reviews on tourism carrying capacity.

References	Research Perspective/Scope	Research Methodology	Main Content
Zhang, Guanghai et al. [5]	TCC Tourism Carrying Capacity	Descriptive analysis	The article reviews the relevant research fields of the concept and application of TECC theory at home and abroad, as well as the problems and trends of the research, and provides an outlook on the research of TECC of urban clusters in China. The evolution of the concept and measurement model for TECC since the 1980s was reviewed.
Li Chen et al. [6]	China’s TCC Tourism Carrying Capacity	Descriptive and statistical analyses	The article reflects on the development and problems of TECC research in China during the 25 years from 1983 to 2008 from both theoretical and practical perspectives, and provides a bibliometric statistical analysis of the categories of TECC publications during this period.
Butler [7]	Geographical perspective	Descriptive analysis	This paper reviews the key elements of the relationship between tourism and the environment, in particular the impact of tourism development on the environment from a geographical perspective, and outlines the difficulties in monitoring environmental change and managing the impact of tourism in destination areas.
Coccossis and Mexa [8]	Sustainable tourism and TECC	Descriptive analysis	This book review states the book regarding TECC. Including the measuring and assessing the carrying capacity of European tourist destinations(United Kingdom, Netherlands, Italy and Croatia), TECC indicator and sustainable tourism indicator.
Saarinen [9]	Sustainable tourism and TECC	Descriptive analysis	The article discusses sustainable tourism in terms of definitions, growth limits, and reviews research on TECC and sustainable development based on the tradition of resources, activities and communities, and proposes a vision for sustainable tourism development under limited conditions.
Pullman and Rodgers [10]	Carrying capacity management for hospitalityand tourism	Descriptive analysis	The article reviews current approaches to carrying capacity management applicable to hospitality and tourism businesses, distinguishes between long-term and short-term strategies in carrying capacity management, and in doing so reviews current research and measurement methods, highlighting the different approaches to capacity management from which various leisure industries can benefit.
Zhong et al. [11]	Environmental impact of tourism in China	Descriptive and statistical analyses	The article summarizes the progress of research on the environmental impacts of tourism in China, particularly reviewing research on the impacts of tourism on the biophysical and socio-cultural environments, tourism carrying capacity, environmental quality assessment, and conservation and management measures of tourism resources. It also proposes that future research should focus on the development of methods to assess the carrying capacity.
Liu, Jia et al. [12]	Tourism Environmental Carrying Capacity Warning	Descriptive analysis	The article introduces the early warning theory and draws on the results of early warning practice in related fields, summarizes the research results of TECC early warning theory and application, and proposes to continuously enrich and improve the content system of TECC research in the future research, and strengthen the research of TECC dynamic early warning and its spatial difference early warning.
Huang et al. [13]	A comparison of tourism carrying capacity studies between China and the UK	Descriptive analysis	The article compares articles based on the environmental impacts of tourism in Chinese and English, especially studies related to carrying capacity and physical impacts. It is found that China lags behind the Western world in this area of research.
Wei et al. [14]	City Carrying Capacity UCC	Descriptive analysis	The article summarizes the literature on urban carrying capacity, integrates the theoretical concepts of urban carrying capacity, compares the advantages and disadvantages of various research methods, and proposes suggestions for urban planning managers to improve UCC.
Marion et al. [15]	Tourism carrying capacity and ecological sustainability	Descriptive analysis	The article summarizes the evolution of recreation ecology research on wilderness environments from 1978–2015, from carrying capacity to visitor use management frameworks, noting the implications of its research for the management of ecological corridors, recreation sites, and wildlife impacts
Li-Yuan et al. [16]	Tourism Psychological Carrying Capacity	Descriptive analysis	The article compares the current situation of domestic and foreign scholars’ research on tourism psychological capacity in scenic areas from 2008–2017 in terms of the concept definition, assessment index, measurement method, influencing factors and response strategies, and proposes to strengthen the database platform construction of simulation experiments and introduce the latest technical means of 3D virtual reality and tourist behavior research for tourism psychological capacity research.
Yang, Xiuping et al. [17]	Tourism carrying capacity	Descriptive analysis	The article compares the relevant researches of scholars since 1960s from five aspects, including the concept of TECC, index system, measurement model, application research and management tools, and proposes that the scientificity and accuracy of TECC measurement model should be improved, and the index system should be constructed from the perspectives of supply index of TECC and demand elements of tourism environment by subjects.
Giglio et al. [18]	Recreational diving and TECC	Descriptive and statistical analyses	The article reviews 67 articles on recreational diving and carrying capacity from 1991–2018 as a way to verify the impact of different recreational diving characteristics on carrying capacity, and concludes with three main challenges facing recreational diving management.
Li et al. [19]	Tourism carrying capacity	Bibliometric analysis	Based on Citespace, the article analyzes the issuing institutions, countries and cooperation networks, co-citation networks and emergent keywords of articles included in WOS on TECC between 2001 and 2020, and identifies five hot research topics and related knowledge gaps.

## Data Availability

The data presented are available upon request from the corresponding author.
